# Serotonin-immunoreactivity in the ventral nerve cord of Pycnogonida – support for individually identifiable neurons as ancestral feature of the arthropod nervous system

**DOI:** 10.1186/s12862-015-0422-1

**Published:** 2015-07-10

**Authors:** Georg Brenneis, Gerhard Scholtz

**Affiliations:** Humboldt-Universität zu Berlin, Institut für Biologie/Vergleichende Zoologie, Philippstraße 13, 10115 Berlin, Germany

**Keywords:** Sea spiders, Neuroanatomy, Neurophylogeny, Evolution, 5-hydroxytryptamine, Immunohistochemistry, *Pycnogonum litorale*, *Meridionale*, *Cilunculus japonicus*

## Abstract

**Background:**

The arthropod ventral nerve cord features a comparably low number of serotonin-immunoreactive neurons, occurring in segmentally repeated arrays. In different crustaceans and hexapods, these neurons have been individually identified and even inter-specifically homologized, based on their soma positions and neurite morphologies. Stereotypic sets of serotonin-immunoreactive neurons are also present in myriapods, whereas in the investigated chelicerates segmental neuron clusters with higher and variable cell numbers have been reported. This led to the suggestion that individually identifiable serotonin-immunoreactive neurons are an apomorphic feature of the Mandibulata. To test the validity of this neurophylogenetic hypothesis, we studied serotonin-immunoreactivity in three species of Pycnogonida (sea spiders). This group of marine arthropods is nowadays most plausibly resolved as sister group to all other extant chelicerates, rendering its investigation crucial for a reliable reconstruction of arthropod nervous system evolution.

**Results:**

In all three investigated pycnogonids, the ventral walking leg ganglia contain different types of serotonin-immunoreactive neurons, the somata of which occurring mostly singly or in pairs within the ganglionic cortex. Several of these neurons are readily and consistently identifiable due to their stereotypic soma position and characteristic neurite morphology. They can be clearly homologized across different ganglia and different specimens as well as across the three species. Based on these homologous neurons, we reconstruct for their last common ancestor (presumably the pycnogonid stem species) a minimal repertoire of at least seven identified serotonin-immunoreactive neurons per hemiganglion. Beyond that, each studied species features specific pattern variations, which include also some neurons that were not reliably labeled in all specimens.

**Conclusions:**

Our results unequivocally demonstrate the presence of individually identifiable serotonin-immunoreactive neurons in the pycnogonid ventral nerve cord. Accordingly, the validity of this neuroanatomical feature as apomorphy of Mandibulata is questioned and we suggest it to be ancestral for arthropods instead. The pronounced disparities between the segmental pattern in pycnogonids and the one of studied euchelicerates call for denser sampling within the latter taxon. By contrast, overall similarities between the pycnogonid and myriapod patterns may be indicative of single cell homologies in these two taxa. This notion awaits further substantiation from future studies.

**Electronic supplementary material:**

The online version of this article (doi:10.1186/s12862-015-0422-1) contains supplementary material, which is available to authorized users.

## Background

Over the centuries, a vast number of neuroanatomical studies have yielded considerable details on arthropod nervous system architecture (e.g. [1–4]). Moreover, the last fifteen years have furthered deeper insights into the cellular basis of neurogenesis and its underlying genetic program in many arthropods other than insects and malacostracan crustaceans (e.g. [5–9]). This has allowed assessing similarities and differences between the four major arthropod groups (Chelicerata, Myriapoda, Hexapoda and paraphyletic crustaceans), ranging from the gross anatomical level all the way down to single identified cells. Notably, some of the findings have provided strong arguments in the ongoing debate on their phylogenetic relationships (e.g. [3, 10, 11]).

In general, the arthropod central nervous system (CNS) has a basic segmental organization [2], being formed by neuromeres, i.e., segmental units of developing neural tissue [12]. These neuromeres give rise to the adult ganglia, whereby each ‘typical’ segmental ganglion is (among others) characterized by (1) a central neuropilar core surrounded by a cortex of the neural somata, (2) segmental nerves targeting an appendage pair (if present), and (3) commissural tracts between the neuropilar cores of the two body halves.

By now, single stereotypic neurons have been individually identified in the ventral nerve cord (VNC) of several arthropod taxa [11, 13–19]. The identification of such stereotypic neurons is most commonly based on soma position and size, neurite morphology and target, use of specific neurotransmitters, developmental origin and gene expression, or sub-sets of these criteria (see [20]). Interestingly, comparisons across arthropods have revealed most similarities between identified neurons of hexapods and crustaceans, especially malacostracans (e.g. [11, 15, 21, 22]), which lends morphological support to the nowadays widely accepted Tetraconata hypothesis [23].

The different neuron types evaluated against a phylogenetic background include the serotonin-immunoreactive neurons of the VNC (e.g. [23–26]). These neurons have been considered promising candidates for ‘homology hunting’ [27], owing to their generally low number per segmental neuromere/ganglion, which facilitates comparisons at the single cell level. Today, serotonin-immunoreactivity has been investigated in the VNC of almost all major arthropod groups, including their close relatives Onychophora [28, 29] and Tardigrada [30, 31]. Notably, the number of serotonin-immunoreactive neurons has been found to be distinctly lower in hexapods and most studied crustaceans, as compared to myriapods and chelicerates. In the first two groups, up to four well-characterized neurons, often arranged in an anterior and a posterior pair, are present per ventral hemi-ganglion [24, 32–38]. In myriapods, on the other hand, between nine and twelve stereotypically arranged segmental somata were detected, yet their neurite morphology remains largely unresolved [25]. Contrasting to this, the studied chelicerates show no indications for singly identifiable serotonin-immunoreactive neurons but instead neuron clusters with often variable and distinctly higher cell numbers (e.g. [25, 39]). Therefore, it has been suggested that the segmental set of serotonin-immunoreactive neurons has undergone a step-wise simplification during arthropod evolution and individually identifiable neurons have been proposed as an evolutionary novelty of myriapods and tetraconates (in support of the Mandibulata hypothesis) (e.g. [10, 40]). However, this scenario hinges on a reliable reconstruction of the ancestral pattern of serotonin-immunoreactive neurons in the chelicerate lineage.

In order to contribute to this, we investigated for the first time serotonin-immunoreactivity in the VNC of three species of Pycnogonida, or sea spiders. The phylogenetic affinities of these exclusively marine arthropods have been intensely debated since their first description (see [41]). Nowadays, they are most plausibly placed within the Chelicerata, as sister group to all other chelicerate taxa, although some authors still favor them as sister group to all other arthropods (=Cormogonida hypothesis) (see [42] for discussion). Irrespective of which hypothesis eventually prevails, investigation of sea spiders is indispensible for the reconstruction of plesiomorphic neuroanatomical features of the chelicerate – if not even the arthropod – stem species. Accordingly, our study aimed to test whether variable clusters of serotonin-immunoreactive neurons represent indeed a plausible ancestral feature of the arthropod VNC. Our results challenge this view. We demonstrate the presence of a sub-set of individually identifiable serotonin-immunoreactive neurons in pycnogonid walking leg ganglia, which show in their arrangement within the ganglionic cortex more overall similarities to the myriapod than to the euchelicerate pattern. This strongly indicates that the VNC of the arthropod stem species was already equipped with segmental sets of individually identifiable serotonin-immunoreactive neurons. Future studies especially on myriapods will help to resolve whether some of the pycnogonid neuron types identified by us can be confidently homologized to neurons present in the mandibulate lineage.

## Methods

### Specimen obtainment and fixation procedures

**Fig. 1:**
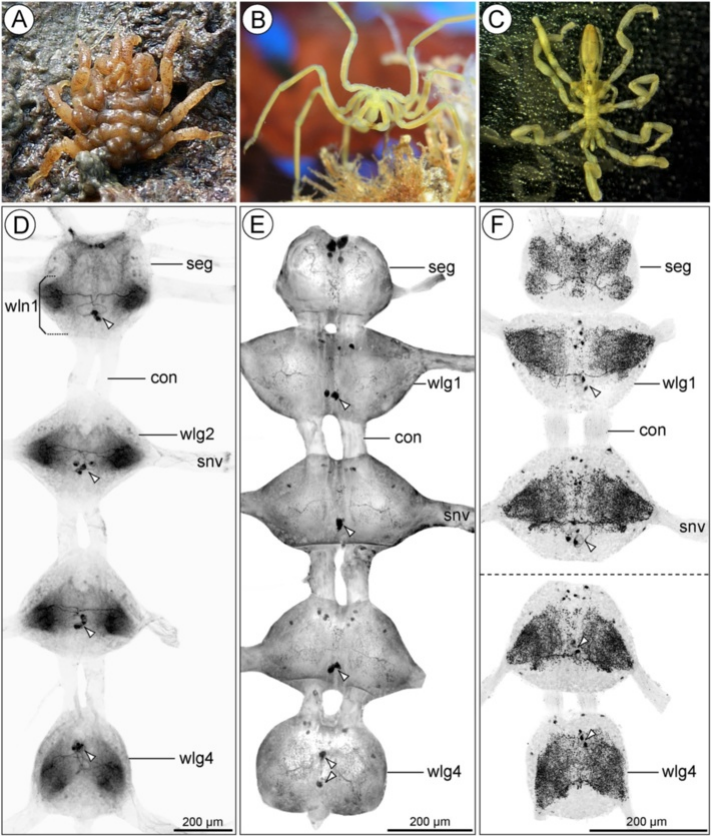
Investigated pycnogonid species and gross morphology of the VNC with SLI overview. **a**: *Pycnogonum litorale*, dorsal view of copulating couple with male clinging to the back of the female. **b**: *Meridionale* sp., anterior view (Original: Claudia Arango). **c**: *Cilunculus japonicus*, dorsal view of fixed specimen. **d-f**: Dissected VNCs of the investigated species in dorsal view, giving an overview of the general SLI pattern. SLI shown in inverted b/w images for better contrast. Arrowheads mark the conspicuous DMN somata in the walking leg ganglia. **d**: *P. litorale*. Note that walking leg neuromere 1 is fused with the palpal and ovigeral neuromeres in an extended subesophageal ganglion. **e**: *Meridionale* sp. Note weaker labeling of the ganglionic neuropil, which is additionally obscured by strong unspecific labeling of the surrounding neural sheath (compare Fig. 4b). **f**: *C. japonicus*. The overview has been composed of two separate images (*dashed horizontal line*)

Adult and sub-adult specimens of *Pycnogonum litorale* (Ström, 1762) (Fig. 1a) were collected in the North Sea near Wilhelmshaven, Germany, and kept in sea water aquaria in the laboratory in Berlin. Husbandry conditions were adopted from previous successful laboratory cultures of *P. litorale* [43–45]. Adult individuals of *Meridionale* sp. (Fig. 1b) were collected in coastal waters near Eaglehawk Neck, Tasmania (see [46, 47]), and briefly kept in the sea water aquaria in Berlin. Specimens of both species were fixed overnight at room temperature in PFA/SW (16 % formaldehyde in ddH_2_0 (methanol-free, Electron Microscopy Sciences, #15710) diluted 1:4 in artificial sea water). The next day, they were transferred to PBS (1.86 mM NaH_2_PO_4_, 8.41 mM Na_2_HPO_4_, 17.5 mM NaCl; pH 7.4) and the CNS was manually dissected, using sharpened forceps (Dumont 5) and electrochemically etched tungsten tips. In order to facilitate antibody penetration of the prominent neural sheath and connective tissue that closely attaches to the CNS of these two species, three to five short pulses in a bath-ultrasonicator (35 kHz, Elma® Transsonic 310) were applied, followed by exposure to a mixture of collagenase and hyaluronidase (Sigma, #C0130 and #H3884, respectively; each at 1 mg/ml in PBS) for 1 h at 37 °C.

Adults of *Cilunculus japonicus* (Turpaeva, 1990) (Fig. 1c) were provided by Günther Pass, University of Vienna, Austria, who in turn obtained them from Katsumi Miyazaki (Kyoto University, Japan). Specimens were fixed for 30 min in 4 % PFA/PBS, the CNS being subsequently manually dissected.

### Immunohistochemistry and fluorescent histochemistry

Prior to antibody exposure, samples were blocked for at least 1 h in PBTx + N (PBS containing 0.5 % bovine serum albumine, 0.3 % Triton X-100, 1.5 % dimethylsulfoxide, and 5 % Normal Goat Serum) at room temperature. Primary and secondary antibodies were diluted in PBTx + N, incubation times lasting at minimum overnight, being sometimes extended up to 48 h at 4 °C. Labeling of serotonin-immunoreactive structures was accomplished with a primary polyclonal rabbit antiserum (Immunostar, #20080, dilution 1:1000) and an Alexa 488-coupled secondary goat antibody (anti-rabbit IgG (H + L), Invitrogen Molecular Probes®, #A11038, dilution 1:400 – 1:500). Although no cross-reactivity was detected by the manufacturer, partial binding of primary antibodies to non-target epitopes cannot be completely ruled out. Therefore, we will cautiously refer to serotonin-like immunoreactivity (SLI) and serotonin-like immunoreactive (SL-ir) structures throughout the text. Labeling of acetylated α-tubulin was performed with a primary monoclonal mouse antibody (anti-ac-α-tub IgG 2b Isotype, clone 6–11 B-1, Sigma T6793, dilution 1:100) and a Cy-3-coupled secondary goat antibody (anti-mouse IgG (H + L), Jackson/Dianova, dilution 1:200). Antibody incubations were every time followed by thorough rinsing in PBTx (block solution without the NGS component, at least 4 h at room temperature) on a horizontal shaker (NeoLab® DOS-20S, 55–70 rpm). Omission of the primary antibodies resulted in complete loss of signal.

Staining of walking leg ganglia with the lipophilic marker FM 1-43FX (Invitrogen Molecular Probes®, #F35355, 5 μg/ml in ddH_2_O) lasted overnight at 4 °C. Nuclear counterstaining was performed with Hoechst (H33342, Invitrogen Molecular Probes®, #H1399, 1 μg/ml in PBS) following preceding labeling procedures. Hoechst incubation lasted at least 1 h and was occasionally extended overnight at 4 °C. After final washing, samples were transferred into Vectashield® Mounting Medium (Vector Laboratories, Inc.) with tiny pieces of plasticine attached to the corners of cover slips acting as spacers.

### Documentation and analysis of fluorescent stainings

Overview images of complete CNSs were taken with a Zeiss Lumar V12, z-stacks being created with Zeiss AxioVision software (Version 4.7.10) and subsequently merged to a single image with extended depth of field using Heliconsoft Helicon Focus software (Version 4.50).

For details, walking leg ganglia were separately scanned with a Leica DM IRE2 confocal laser-scanning microscope equipped with a Leica TCS SP2 AOBS laser-scanning unit, step sizes ranging from 0.4 to 2.0 μm between successive scanning planes, depending on the intended z-resolution. Subsequent analyses were performed with the 3D-reconstruction program ‘Imaris’ (Bitplane AG, Switzerland, version 7.0.0.). Neurites were individually traced in 3D volumes (Surpass mode) as well as in the Section mode, in which all three optical section planes are shown and navigated simultaneously. In order to depict the three-dimensionally extending neurites of individual SL-ir neurons as completely as possible, snapshots taken in Imaris do often not represent single optical sections, but rather Imaris volumes of the scanned image stacks (Maximum intensity projection), to which multiple clipping planes have been applied. In *Meridionale* sp., the serotonin-immunolabeling resulted in a strong unspecific signal of the neural sheath. To visualize the SL-ir structures within the ganglia more clearly, this unspecific labeling was masked by manual segmentation. Supplementary movies were generated in Imaris (Animation module), being subsequently transformed into MP4-format using the freeware FormatFactory (version 2.96, www.pcfreetime.com).

### Histology

Adults of *Meridionale* sp. were fixed in Bouin’s solution (15 parts saturated aqueous picric acid, five parts 37 % formaldehyde (methanol-stabilized), one part glacial acetic acid) for 30–40 min, followed by repeated washing and long-term storage in 70 % ethanol. Specimens were embedded in Technovit 7100 plastic resin (Kulzer Histo-Technik) following the manufacturer’s standard protocols. Semi-thin sections (1.5 μm) were cut with a Microm HM 355 microtome, stretched at 60 °C on a heating plate and stained in a first step with methylene blue-azure II, followed by a counterstain in basic fuchsine solution. Sections were embedded in Roti®-Histokitt (Roth) under cover slips. Photographs of selected sections were taken with a Zeiss Axioskop 2 plus microscope equipped with a digital camera (Zeiss AxioCam HRc).

### Data presentation

The descriptions of the SL-ir neuron types refer to one body half only, deviations from this are explicitly mentioned. Global contrast and brightness values of some of the images were adjusted using Adobe Photoshop CS3. Images depicting only SL-ir structures were converted into inverted black-and-white images for enhanced contrast. All figures were compiled in Adobe Illustrator CS3. If not stated otherwise in the figure legends, anterior is to the top in all ventral or dorsal aspects and in horizontal sections. In anterior or posterior aspects and transverse sections, dorsal is to the top.

## Results

### Ventral nerve cord

The VNC of pycnogonids is composed of a chain of separate ganglia that are longitudinally linked by paired connectives (Fig. 1d-f). In *Cilunculus japonicus* and *Meridionale* sp., the anterior-most part of the VNC is the subesophageal ganglion, which is a composite structure formed by the palpal and ovigeral neuromeres during embryonic development. It is followed by four separate walking leg ganglia (Fig. 1e,f). In contrast to this, *Pycnogonum litorale* has an ‘extended’ subesophageal ganglion, which encompasses not only the palpal and ovigeral neuromeres but also walking leg neuromere 1. However, despite this fusion in *P. litorale*, the neuropil of walking leg neuromere 1 remains easily distinguishable from the anteriorly adjacent ovigeral one (Fig. 1d). This permits clear segmental assignment of SL-ir neuronal somata based on their position in the cortex and the course of their projections into the respective neuropil regions. The SL-ir neurons in walking leg neuromere 1 of *P. litorale* could therefore be directly compared with those in the corresponding walking leg ganglia of *Meridionale* sp. and *C. japonicus*.

### Gross architecture of a pycnogonid walking leg ganglion

**Fig. 2:**
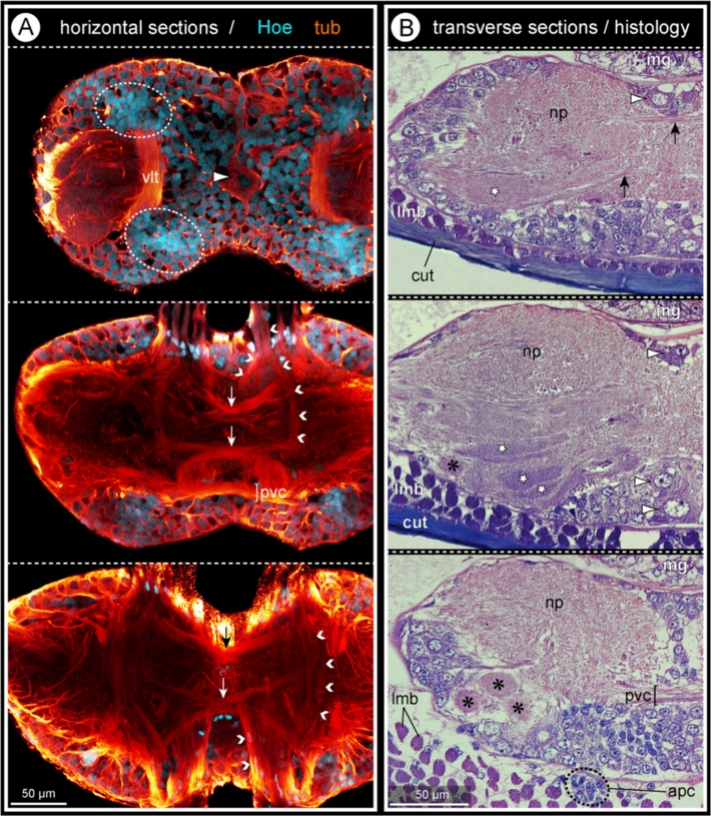
Architectural features of pycnogonid walking leg ganglia exemplified by *Meridionale* sp. **a**: Optical horizontal sections through walking leg ganglion 1, proceeding from ventral (*uppermost*) to farther dorsal; tubulin labeling (*glow*) with Hoechst counterstain (*cyan*). Note densely packed small nuclei of globuli-like cells (*stippled ovals*) in the anterior and posterior portions of the cortex. The large arrowhead marks a large cell soma in the midline region of the ganglion. Small arrowheads indicate different longitudinal neurite bundles passing through the neuropilar core. White arrows highlight chiasm-like transverse tracts, black arrow an antero-dorsal commissural tract. Note the slightly set off postero-ventral commissural tract (pvc). **b**: Transverse histological sections through walking leg ganglion 2, proceeding from anterior (*uppermost*) to farther posterior; midline region at the right side. Selected elongate and ovoid glomerulus-like neuropils are marked in the ventral ganglion half (stars and asterisks, respectively). Arrowheads indicate large cell somata in the midline region. Black arrows indicate commissural tracts

Apart from the dorsal side, a somata cortex encloses the medially fused neuropil halves of each walking leg ganglion (WLG) (Fig. 2b). Especially in the midline region, conspicuously large somata with a copious cytoplasmic compartment are found (Fig. 2a,b), whereas regions of densely packed small somata – reminiscent of globuli cells – are positioned antero-ventral and postero-ventral to the neuropil in each hemiganglion (Fig. 2a).

**Fig. 3:**
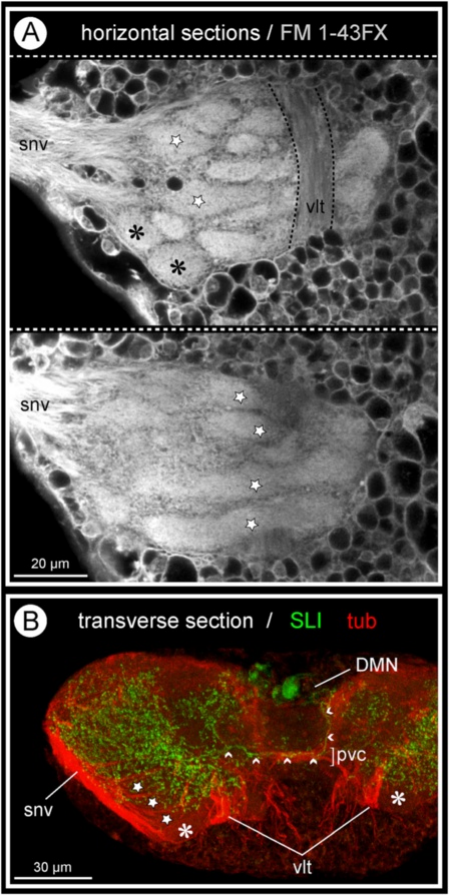
Architectural features of pycnogonid walking leg ganglia exemplified by *C. japonicus.* **a**: Optical horizontal sections through walking leg ganglion 1 labeled with lipophilic marker FM 1-43FX. Uppermost image farther ventral than lower one. Selected elongate and ovoid glomerulus-like neuropils are marked (stars and asterisks, respectively). **b**: Optical transverse section through walking leg ganglion 3, tubulin labeling (*red*) and SLI (*green*). Selected elongate and ovoid glomerulus-like neuropils are marked in the ventral lobe (stars and asterisks, respectively). Note neurite bundles that branch off the root of the segmental nerve and project into the ventral lobe with its glomerulus-like neuropils. Small arrowheads highlight the contralaterally extending DMN neurite in the postero-ventral commissure

**Fig. 4:**
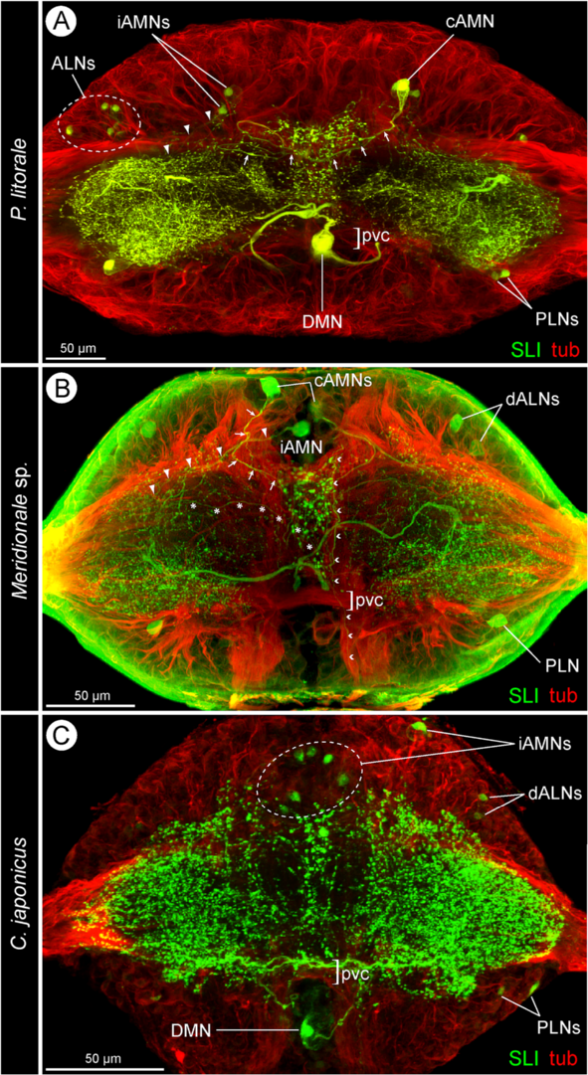
General SLI pattern in pycnogonid walking leg ganglia. **a-c**: Extended horizontal sections through walking leg ganglion 2 of the investigated species, showing sub-sets of the identified SL-ir neurons. Imaris Surpass mode with applied clipping planes (**a,** **c**) or Imaris Section mode (**b**), tubulin (red) and SLI (green). Large arrowheads indicate course of ipsilateral iAMN neurites. Arrows follow the loop of contralaterally projecting cAMN neurites towards the median SL-ir domain. **a**: *P. litorale*. The intensely labeled neurites of the DMNs (only one DMN soma included in the section) pass the postero-ventral commissure dorsally and proceed anterior to it into the contralateral neuropil. **b**: *Meridionale* sp. Note the unspecific green labeling of the neural sheath, which is only observed in this species. Small arrowheads mark a longitudinal SL-ir axon passing through connectives and the walking leg ganglion. The soma of one cAMN is not fully included in shown section. Asterisks mark the slender contralateral neurite of a small DMN (soma not in section). Note the massive contralateral neurites of the large DMNs (somata not in section) passing directly anterior to the postero-ventral commissure. **c**: *C. japonicus*. The contralaterally projecting DMN neurites (one soma included in section) cross the midline as part of the postero-ventral commissure

Numerous separate commissural tracts of varying diameter cross the midline within the neuropil, being located at different levels along the antero-posterior as well as the dorso-ventral axes (Fig. 2a,b). Among them are several chiasm-like structures (Fig. 2a). Set off from the posterior neuropil margin, a distinct postero-ventral commissure is present in all three investigated species (Figs. 2a,b; 3b and 4). The densely packed axon bundles of the connectives separate upon entering the ganglionic neuropil, some proceeding at its dorsal or ventral surface, others passing through its center (Figs. 2a; 3a,b).

In the dorsal half of the ganglion, no specific organization of the neuropil is discernible (Fig. 2b). By contrast, the ventral neuropil portion forms a lobe that protrudes into – and in some places even breaches through – the somata cortex, extending to the ventral ganglion surface (Fig. 2b). This lobe contains elongate and ovoid-shaped glomerulus-like neuropils (GLNs), which are targeted by afferents from the segmental nerve (Figs. 2b; 3a,b).

### SLI in the walking leg ganglia

**Fig. 5:**
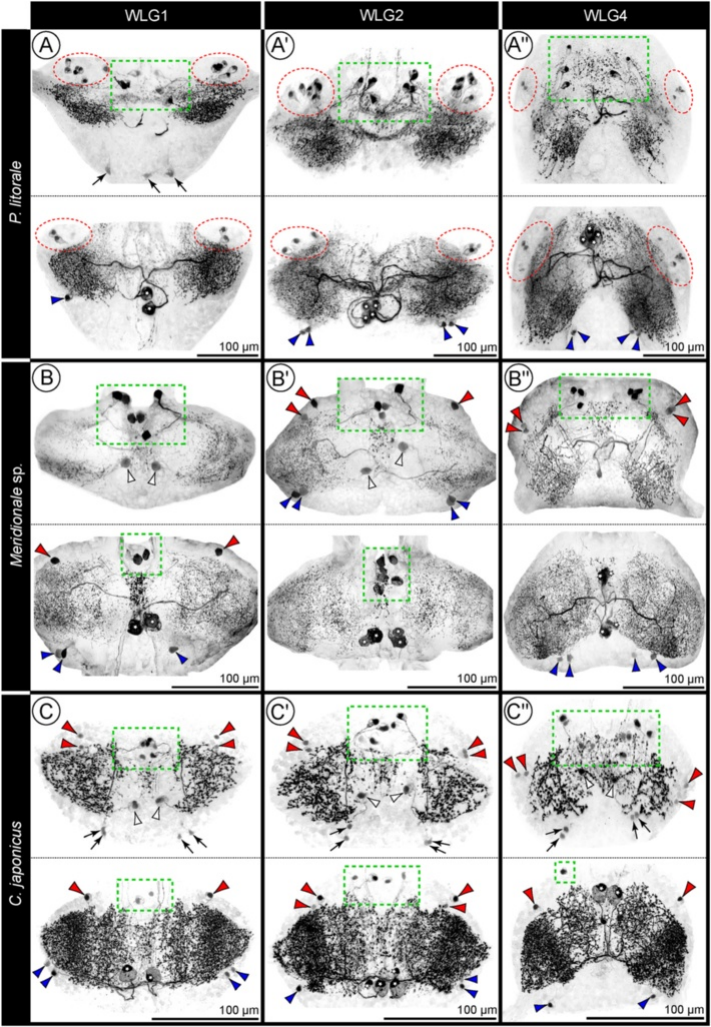
Comparison of SL-ir structures in the walking leg ganglia 1, 2 and 4 of the investigated pycnogonids. SLI signal shown in inverted b/w images for better contrast. Each of the depicted three walking leg ganglia has been roughly divided into a ventral and a dorsal half (upper and lower image, respectively). This facilitates intra- and inter-specific comparisons, since overlapping of separate neuron types along the dorso-ventral axis is reduced. White stars mark the DMN somata. Green dashed rectangles indicate the AMN somata. Red arrowheads and red stippled ovals highlight the ALN somata. Blue arrowheads point at the PLN somata. White arrowheads mark the VMN somata. Black arrows show the PVN somata. **a-a''**: *P. litorale*. **b-b''**: *Meridionale* sp. **c-c''**: *C. japonicus*

**Fig. 6:**
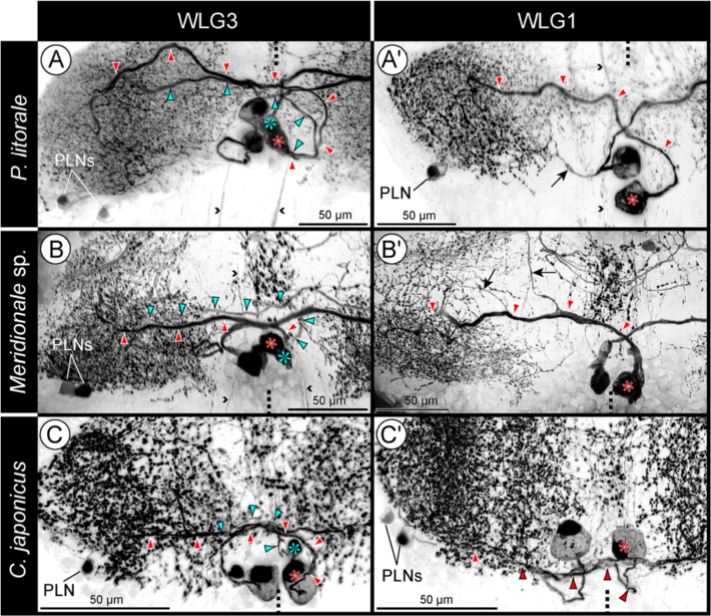
Comparison of DMNs in WLG1 and WLG3 of the investigated pycnogonids. SLI signal shown in inverted b/w images for better contrast. In order to reveal as many details of the neurites as possible, clipping planes have been applied to Imaris volumes. Dashed lines indicate midline region. Small arrowheads indicate selected longitudinal SL-ir axons within the connectives and passing through the ganglionic neuropil. Note that in case of two labeled PLNs in the shown volume, one soma shows weaker signal intensity. **a-c**: Walking leg ganglion 3. Each ganglion features two unipolar DMNs per hemiganglion, their respective primary neurite projecting contralaterally (red/light blue asterisks and arrowheads). With the exception of *P. litorale* (**a**), one DMN has a smaller soma and more slender neurite. **a’-c’**: Walking leg ganglion 1. Only one large DMN is present in each hemiganglion (red asterisk and arrowheads). **a’** shows an exceptional case, in which a slender ipsilateral projection (*arrow*) branches off the contralaterally extending primary neurite of a DMN. **b’** shows two anterior projections (*arrows*) send out from the further posteriorly extending contralateral DMN neurite

**Fig. 7:**
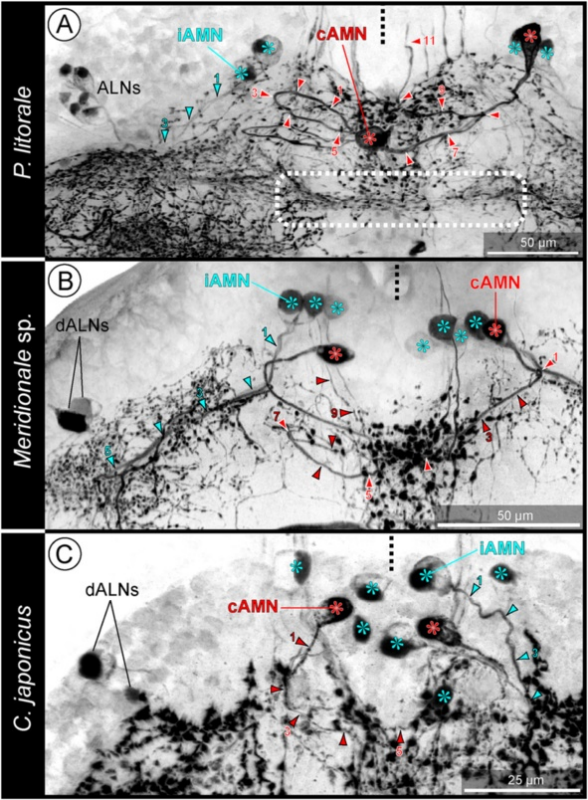
Comparison of cAMNs and iAMNs in WLG2 of the investigated pycnogonids. SLI signal shown in inverted b/w images for better contrast. In order to reveal as many details of the neurites as possible, clipping planes have been applied to Imaris volumes. Dashed lines indicate midline region. Red asterisks highlight cAMN somata, red arrowheads (numbered) illustrate the course of the primary neurite of the cAMN of one body half. Corresponding light blue markings have been used of the iAMNs. **a**: *P. litorale*. The soma of the right cAMN is in an unusually far lateral position. The dashed white rectangle highlights the lateral extensions that interconnect the SL-ir median domain with the more lateral SL-ir domains in each hemiganglion. **b**: *Meridionale* sp. Note the weakly stained somata of additional iAMNs. **c**: *C. japonicus*. Owing to the dense neuropilar meshwork in the median domain it is challenging to follow the delicate contralateral neurite of the cAMN (see also Additional file 4)

**Fig. 8:**
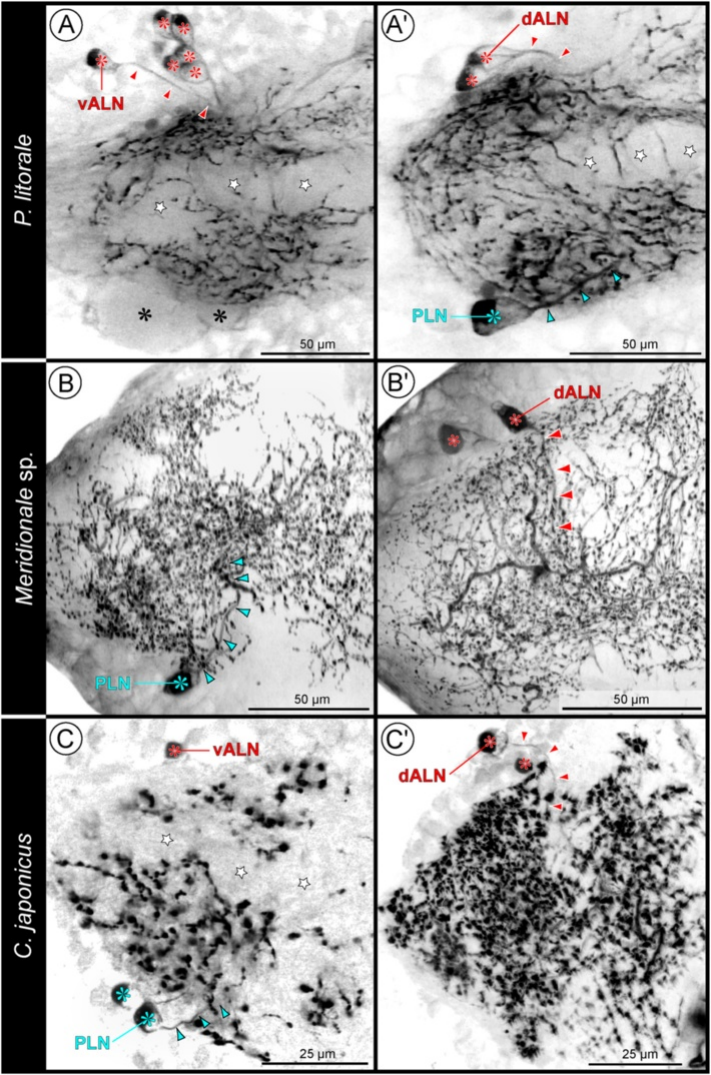
Comparison of ALNs and PLNs in WLG2 of the investigated pycnogonids. SLI signal shown in inverted b/w images for better contrast. One hemiganglion shown, the midline region is located towards the right. In order to reveal as many details of the neurites as possible, clipping planes have been applied to Imaris volumes. Images to the left (**a**-**c**) depict ganglion regions further ventral than corresponding images to the right (**a’**-**c’**). Red asterisks highlight ALN somata, red arrowheads illustrate the course of the primary neurite of one of the ALNs. Corresponding blue markings have been applied to the PLNs. Stars and black asterisks mark signal-free regions of elongate and ovoid glomerulus-like neuropils, respectively. **a,a’**: *P. litorale*. Note the high number of ALNs, the somata of which being spread out in a loose cluster-like array from ventral to dorsal. **b,b’**: *Meridionale* sp. The second PLN with comparable neurite course is not visible in the section shown in **b. c,c’**: *C. japonicus*

Each walking leg hemiganglion contains a dense meshwork of SL-ir dendritic arborizations and axon collaterals that extends almost through its entire neuropilar core (Figs. 1d-f; 3b; 4; 5; see also Additional files 1, 2 and 3). This SL-ir meshwork engulfs also the elongate and ovoid GLNs of the ventral lobe. However, while it penetrates into their outer layers, the inner cores of the GLNs are almost devoid of signal. (Fig. 3b; see also Fig. 8a,a’,c). In the midline region, an unpaired antero-posteriorly elongated SL-ir domain is recognizable, being laterally defined by some of the longitudinal tracts that pass through the ganglionic neuropil (e.g. Figs. 4; 6b-c’; Additional file 4). Especially in *P. litorale*, this median domain has distinct transverse extensions, which connect it with the SL-ir domains in the hemiganglia (Figs. 4a; 5a,a’; 7a; Additional file 1). To all appearances, these transverse extensions are predominantly of neuropilar nature; they do not include any of the tubulin-positive commissural tracts, nor do they show any continuous SL-ir axonal projections within.

The somata of the SL-ir neurons are arranged in characteristic positions within the cortex (Figs. 4; 5). The neurons will be described – and where possible individually identified – according to their number, soma position and neurite morphology (see below). Almost all of them were found to be unipolar – merely in some specimens *P. litorale*, a bipolar morphology of one neuron type was occasionally but not consistently encountered (Additional file 5; see below). An overview of all encountered SL-ir neuron types is presented in Table 1.

**Table 1:** Serotonin-like immunoreactive neurons in walking leg ganglia of the investigated pycnogonid species

SL-ir neuron type				*Pycnogonum litorale* (*N* = 7)	*Meridionale* sp. (*N*= 3)	*Cilunculus japonicus* (*N* = 6)
Abbreviation	Soma position	Primary neurite	Neurite course & morphology	No. & Comments	No. & Comments	No. & Comments
DMN	medial, dorsal	contralateral	extensive arborizations in contralateral hemiganglion	WLG1: 1 large DMN	WLG1: 1 large DMN	WLG1: 1 large DMN
				WLG2-4: 2 large DMNs;	WLG2-4: 1 large, 1 small DMN;	WLG2-4: 1 large, 1 small DMN;
				strong SLI	strong SLI	strong SLI
cAMN	antero-medial, ventral	contralateral	contributes to median SL-ir domain + projects into contralateral connective	WLG1-4: 1 cAMN; strong SLI;	WLG1-4: 1 cAMN; strong SLI	WLG1-4: 1 cAMN; strong SLI
				bipolar in some WLGs of 3 specimens		
iAMN	antero-medial, ventral to dorsal	ipsilateral	projects far laterally along anterior margin of ipsilateral SL-ir domain	WLG1-4: 2v iAMNs; strong SLI	WLG1: 2v + 1 iAMNs	WLG1: 2v + 1 iAMNs
					WLG2-4: 2v + 2 iAMNs;	WLG2-4: 2v + 2 iAMNs;
					strong SLI	strong SLI
ALN	antero-lateral, ventral to dorsal	ipsilateral	projects posteriorly into ipsilateral SL-ir domain	WLG1-4: 5–14 iAMNs;	WLG1: 1d ALN	WLG1: 1-2v & 1d ALNs
				typically ~10 neurons from v to d;	WLG2-4: 0-2v & 1 or 2d ALNs;	WLG2-4: 1-2v & 1-2d ALNs;
				varying intensity of SLI	vALNs low SLI	vALNs low SLI
PLN	postero-lateral, central to dorsal	ipsilateral	projects anteriorly into ipsilateral SL-ir domain	WLG1: 0 or 1 PLNs	WLG1&4: 1 or 2 PLNs	WLG1&2: 1 or 2 PLNs
				WLG2-4: 0–2 PLNs	WLG2&3: 2 PLNs;	WLG3&4: (0 or) 1 PLN
				if 2 PLNs, 1 often lower SLI	if 2 PLNs, 1 often lower SLI	if 2 PLNs, 1 often lower SLI
VMN	medial, far ventral	ipsilateral	projects postero-laterally into postero-medial region of ipsilateral SL-ir domain	WLG1-4: 0 or 1 VMN; low SLI	WLG1-4: 0 or 1 VMN; low SLI	WLG1-4: 1VMN, low SLI
PVN	postero-medial, ventral	ipsilateral	extends anteriorly into postero-medial region of ipsilateral SL-ir domain	WLG1-4: 0–2 PVNs; low SLI	WLG1-4: −	WLG1-4: 1 or 2 PVNs; low SLI

The number of studied specimens is indicated in brackets. Neuron numbers are given for hemiganglia, i.e. for one body half. Abbreviations: v = ventral; d = dorsal

Within the connectives between the ganglia as well as in the longitudinal tracts within each ganglion, several SL-ir neurites are encountered (e.g. Figs. 4b; 6; 7). The origin of some but not all of those axonal projections could be traced to specific SL-ir somata (see below). Contrasting to this, no SL-ir neurites were detected in the segmental nerves of any of the investigated species.

### Dorso-medial neurons (DMNs)

The DMNs are consistently identifiable, very conspicuous neurons in WLG1-4 (Figs. 1d-f; 5; 6; Additional files 1, 2 and 3). Their somata are placed far dorsal in the cortex, always medial to the connective. Invariably, WLG1 has only a single DMN, whereas WLG2-4 possess two DMNs (Figs. 1d-f; 5; 6). The soma of the single DMN or of at least one of the present two DMNs has a distinctly larger cytoplasmic compartment and thicker primary neurite than all other SL-ir neurons in the respective ganglion. The primary neurites of the DMNs project ventrally before crossing the midline into the contralateral neuropilar core. Within the latter, they show extensive arborizations that extend into the dorsal as well as into the ventral neuropil half. No projections into the connectives could be traced, i.e., the DMNs are very likely local interneurons.

#### Inter-ganglionic pattern variations common in all three species

In WLG1-3, the DMN somata are positioned postero-medially to the ipsilateral SL-ir domain, whereas they are frequently shifted anteriorly in WLG4 (Figs. 1d-f; 5a”,b”,c”). However, this positional shift does not always include all DMN somata (e.g. Figs. 1e; 5b”). The neurites of anteriorly shifted somata extend initially in a posterior direction, but then project contralaterally as in the more anterior WLGs. The deviating DMN somata arrangement of WLG4 might be related to a fusion process in late post-embryonic development, during which cellular material of two additional posterior small ganglion anlagen is incorporated into the posterior cortex region of WLG4 (see [48]).

#### Inter-specific pattern variations

(1) In *Meridionale* sp., the somata of the DMNs are not always spherical or ellipsoid but often deformed in the dense packing of the cortex (e.g. Figs. 5b”; 6b’). Their contralateral arborizations are especially concentrated in the posterior neuropil portion, but some collaterals projecting into the anterior neuropil portion can be also observed (e.g. Fig. 6b’). In *P. litorale* and *C. japonicus*, no comparably pronounced pattern was encountered.

(2) In *P. litorale*, the somata of both DMNs in WLG2-4 are distinctly larger than the remaining SL-ir neurons and possess thick primary neurites (Figs. 5a’,a”; 6a). In this species, the only instance of a slender ipsilateral projection branching off from the contralaterally extending primary neurite was observed in a single WLG (Fig. 6a’).

(3) In *P. litorale* and *Meridionale* sp. the contralaterally projecting neurites of the DMNs pass anterior to the postero-ventral commissure (Fig. 4a,b), whereas they join the latter in *C. japonicus* (Figs. 3b; 4c).

### Antero-medial neurons (AMNs)

Three AMNs are consistently identifiable in WLG1-4 (Figs. 5; 7; Additional files 1, 2, 3 and 4). Their somata are often positioned very close to each other, antero-ventrally in the cortex. In the majority of cases, they lie either medial to, or at the level of the connective, but never far lateral in the ganglion.

Invariably, one of the neurons (cAMN) is an ascending neuron with intensely labeled soma. Its primary neurite projects initially in an ipsilateral direction, then turns in a loop and crosses contralaterally within an antero-ventral commissural tract (Figs. 4a,b; 7; Additional files 1, 2 and 4). On its way, it passes along the anterior border of the median SL-ir domain into which it sends off slender projections that could not be followed in any more detail. Within the contralateral neuropil, the neurite turns into a dorsally directed loop and sends a projection antero-dorsally into the connective (Fig. 7a,b).

The other two consistently labeled neurons (iAMNs) have ipsilaterally extending primary neurites that often fasciculate closely and enter the anterior portion of the ipsilateral SL-ir domain, where they proceed far laterally (Figs. 4a,b; 7). Secure tracing of their subsequent branching pattern was not always possible. In some specimens, however, delicate projections extending into the postero-lateral neuropil region could be observed.

#### Inter-specific pattern variations

1. In *P. litorale*, the cAMN was found to be bipolar in some ganglia in a sub-set of the investigated specimens. These ganglia were not always in the same segment across the different specimens. The soma’s additional slender projections extend directly into the median SL-ir domain (Additional file 5).
2. In *C. japonicus* and *Meridionale* sp., one or two additional SL-ir AMNs are present (Figs. 5b-c”; 7b,c) in WLG1 or WLG2-4, respectively. They feature an ipsilaterally projecting primary neurite with a course comparable to the two consistently labeled ventral iAMNs. Often, their somata are less intensely labeled (e.g. Fig. 7b) and/or positioned further dorsally than those of the latter (e.g. Fig. 5b,b’,c-c”).

### Antero-lateral neurons (ALNs)

This group of neurons shows the highest variation of numbers between the three investigated species, with at least one but also more than 10 ALNs being present in WLG1-4 (see details in inter-specific variations). The somata are located antero-laterally in the cortex, distinctly lateral to the connective (Figs. 4; 5; 7; Additional files 1, 2 and 3). The primary neurite of each ALN extends posteriorly into the lateral portion of the ipsilateral SL-ir domain (Fig. 8a,a’,b’,c’). The course of the delicate projections could be traced in the dense SL-ir neuropilar meshwork only for a relatively short distance. Along this distance, no indications of any contralateral extensions were found.

#### Inter-specific pattern variations

In *Meridionale* sp., a single dorsal ALN was found in WLG1 (Fig. 5b), whereas two dorsal ALNs (one with lower signal intensity) were labeled in the majority of WLG2-4 (Figs. 5b’,b”; 7b; 8b’). In one specimen, two additional but very weakly stained ventral ALNs were observed in some WLGs.

In *C. japonicus*, a single dorsal ALN and two weakly labeled ventral ALNs are found in WLG1 (Fig. 5c). The majority of WLG2-4 show a dorsal pair of ALNs with one soma being stained less intensely (Figs. 5c’; 7c; 8c’) as well as a weakly labeled ventral pair (Fig. 5c-c”; Additional file 4). In some hemiganglia, however, only single ventral or dorsal ALNs could be identified (Fig. 8c and Fig. 5c”, respectively).

In *P. litorale*, the number of ALNs is highest. Being spread out from dorsal to ventral in a loose cluster-like array (Figs. 5a-a”; 7a; 8a,a’), the number of SL-ir somata (with different signal intensities) ranges from minimally five (e.g. Fig. 5a”) to up to 14 per hemiganglion. Most often a number close to 10 is being labeled.

### Postero-lateral neurons (PLNs)

Maximally two PLNs occur in WLG1-4 (Figs. 5; 6; 8; Additional files 1, 2 and 3). Their somata are positioned postero-laterally in the cortex, lateral to the connective and directly adjacent to the ipsilateral SL-ir domain (Figs. 4; 5). Furthermore, they are located dorsal to the ovoid-shaped glomerulus-like neuropils (e.g. Fig. 8a,a’). The primary neurite of a PLN extends at first in a medial direction and soon after projects anteriorly into the SL-ir domain (Fig. 8a’,b,c). Here, it could be followed for a distance in an anterior direction, but beyond that, its course and arborizations could not be unequivocally clarified.

#### Intra-ganglionic pattern variations common in all three species

In deviation from a strictly bilaterally symmetrical pattern, several WLGs featured two labeled PLNs in one hemiganglion, whereas only one PLN could be detected in the other (Fig. 5b,c’; Additional files 1 and 3), rarely not even a single one (e.g. Fig. 5a). Furthermore, in case of two labeled PLNs, one often showed a distinctly lower signal intensity (e.g. Fig. 6a,b,c’). No consistent inter-specific differences were assessable for these variations.

### Ventro-medial neuron (VMN) and postero-ventral neurons (PVNs)

The VMN and PVNs are weakly labeled neurons, which were not consistently detected in all of the three investigated species. The soma of the VMN is located far ventral in the cortex and medial to the ventral lobe containing the glomerulus-like neuropils (Fig. 5b,b’,c-c”; Additional files 3 and 4). Its primary neurite remains ipsilateral, projects postero-laterally and enters the ipsilateral SL-ir domain at its posterior side (Additional file 4). The somata of the up to two PVNs are positioned further postero-ventrally in the cortex (Fig. 5a,c-c”; Additional files 3 and 4), their primary neurites extending anteriorly into the ipsilateral SL-ir domain (Additional file 4).

#### Inter-specific pattern variations

In *C. japonicus*, the presence of the VMN and two PVNs could be confirmed in the majority of investigated WLGs. It is therefore here considered as being a common feature of the pattern in this species. In *P. litorale* and *Meridionale* sp., these two neuron types could be only unreliably identified – in fact, PVNs appear to be completely missing in WLG1-4 of *Meridionale* sp.

## Discussion

### The commissural system in pycnogonid ganglia and other arthropod taxa

Commissural tracts can be used as landmarks that help to characterize identified neurons with contralateral projections in more detail. The fasciculation of a contralaterally projecting neurite with a specific commissural tract provides additional information for homologization with other neurons. In case of the SL-ir neurons in the arthropod VNC, contralateral projections of supposedly homologous neurons have been repeatedly assigned either to *the* anterior or *the* posterior commissure (e.g. [10, 12, 15, 26]). However, such a simple two-commissure-system per segmental neuromere is not consistently found throughout arthropods. On the contrary, it is relatively rare in adult animals (e.g. Branchiopoda [24]; Mystacocarida [49]; Remipedia [26]) and many other taxa exhibit either more complex commissural systems (see [27, 50–53], this study) or the architecture remains still unsatisfactorily resolved (e.g. in Myriapoda). Accordingly, a segmental two-commissure-system is not an ubiquitous arthropod feature and cannot be confidently advocated as plesiomorphic state of the adult VNC.

Similar disparities are encountered during development of the commissural system. Especially in tetraconates, a distinct two-commissure-system is frequently recognizable during segmental axonogenesis [54–56]. But yet again, neither does this apply to all tetraconate representatives (e.g. [16, 57]), nor does it hold for the studied euchelicerates [19, 58] and apparently also not for myriapods [9, 14]. In pycnogonids, there is only a single rather than two embryonic commissures [8, 59] and it is only during post-embryonic development that this primary commissural pathway is further elaborated into the numerous separate transverse tracts of the adult system. Hence, the more complex adult commissural systems of pycnogonids, euchelicerates and supposedly myriapods cannot be convincingly derived from a transient two-commissure system in development, as may be possible for many tetraconates [4, 60].

As a consequence, when identifying and homologizing contralaterally projecting SL-ir neurons in intra-specific comparisons, the exact neurite pathway through specific commissural tracts is beyond doubt very useful. However, whenever dealing with inter-specific neuron homologization across divergent taxa, correspondences between the investigated commissural systems should be ascertained first, before a comparison of the exact course of single contralateral neurite projections makes sense. Strictly speaking, only contralateral projections through corresponding tracts within the compared commissural systems can add additional support to inter-specific neuron identity and thus strengthen a homology hypothesis. In this regard, available data on pycnogonids, euchelicerates and myriapods remain still unsatisfactory, which hampers arthropod-wide comparisons.

### Intra-specific comparison of SL-ir neurons in the pycnogonid walking leg ganglia

**Fig. 9:**
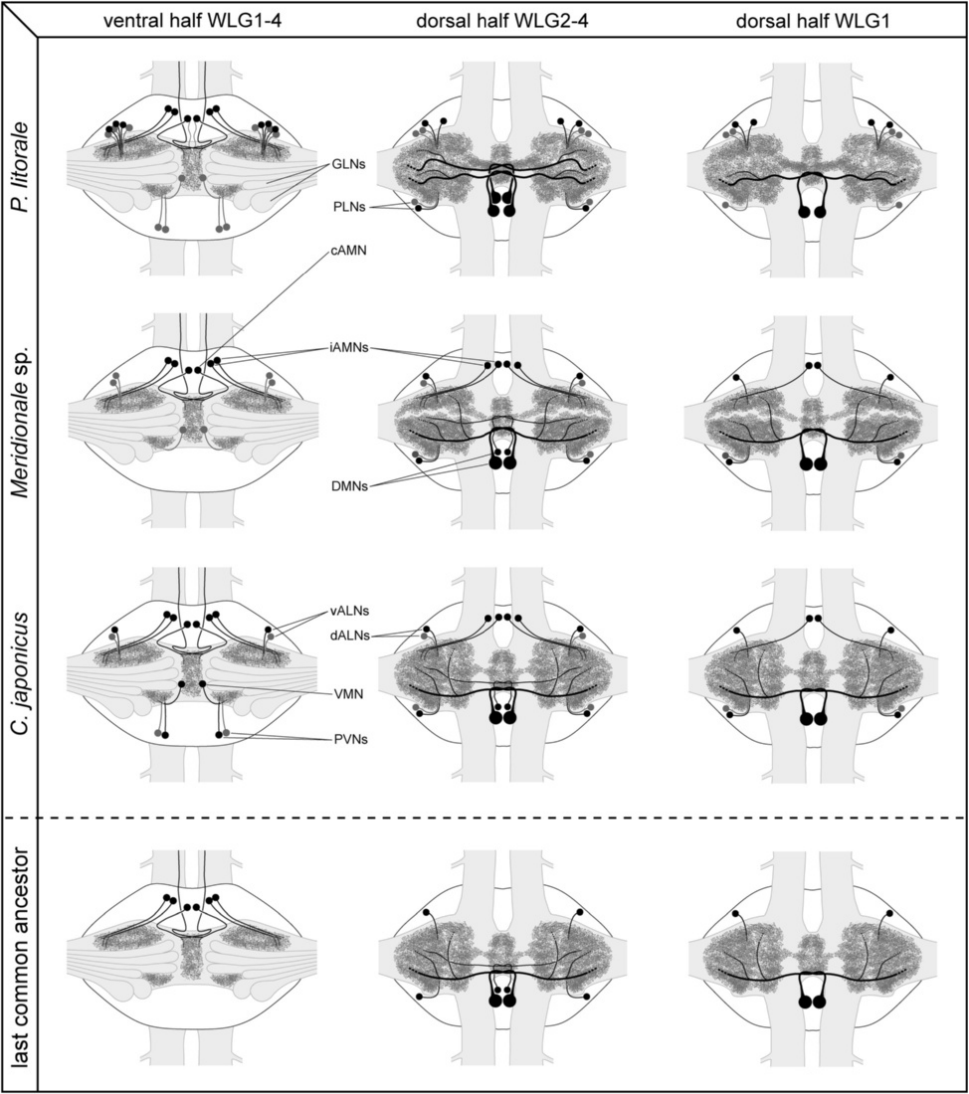
Schematic representation of SLI pattern in the investigated pycnogonids and inferred minimal pattern in the last common ancestor. The left column shows the largely corresponding SLI pattern of the ventral half of all four walking leg ganglia, the middle column depicts the pattern characteristic for the dorsal half of walking leg ganglia 2–4, and the right column for the slightly deviating dorsal half of walking leg ganglion 1 only. All neurons shown in black have been consistently identified in the respective species. Neurons in gray were only inconsistently identified. In the case of the ALNs of *P. litorale*, five neurons are shown in black (minimum number observed) and the additional gray neurons indicate that higher numbers are frequently observed, but no clearly fixed maximum cell number was assessable. The lowest row illustrates the reconstructed minimal pattern of the common ancestor, including only those neuron types and neuron numbers that have been reliably recovered and therefore homologized across the three investigated pycnogonid species

In each of the studied pycnogonid species, different SL-ir neuron types have been reliably identified in the four walking leg ganglia of single individuals as well as across different specimens. Some of these neurons have been found to be intra-specifically strictly stereotypic (DMNs, cAMN, iAMNs), whereas others show variations of cell numbers (ALNs and PLNs) or were not even reliably detected in each species (VMN and PVNs) (Fig. 9; Table 1).

At the moment, it remains unresolved whether the observed intra-specific differences relate to actual variability in the cell number of the respective SL-ir neuron type. This is because several of the neurons show whenever labeled very low signal intensity and we cannot fully exclude that non-detection of them may be related to a less successful immunolabeling. Furthermore, the applied protocol does not allow elucidating whether some of the neurons in question show significant temporal variations in serotonin synthesis and content. This might lead to the detection of only a sub-set of SL-ir neurons of ganglia and individuals (see [61]). Similar uncertainties relating to weakly labeled neurons have been encountered in previous studies (e.g. [26, 62]). However, regardless of the cause of variation in pycnogonids, it is notable that the observed variability is – with the exception of the ALNs in *P. litorale* – restricted to the absence of one or two cells of a specific SL-ir neuron type. It does not relate to neuron clusters of generally higher cell numbers – a feature considered as being typical for euchelicerate taxa (see below).

### Inter-specific comparison of SL-ir neurons and the SLI pattern of the last common ancestor

Beyond intra-specific correspondences, striking inter-specific similarities in terms of soma position and neurite projection patterns are assessable for most of the detected SL-ir neuron types. This holds in particular for the DMNs, cAMN, iAMNs and PLNs, but to some extent also for the ALNs (Fig. 9; Table 1). These correspondences strongly support the homology of the neuron types across the three species.

Interestingly, the inter-specific similarities do even extend to some pattern variations between different WLGs. The most eye-catching instance is the consistent lack of a second DMN in WLG1 as compared to WLG2-4. Likewise, WLG1 of *C. japonicus* and *Meridionale* sp. shows a corresponding pattern of only one instead of two dorsal ALNs, as well as only three instead of four iAMNs (Fig. 9; Tab. 1).

**Fig. 10:**
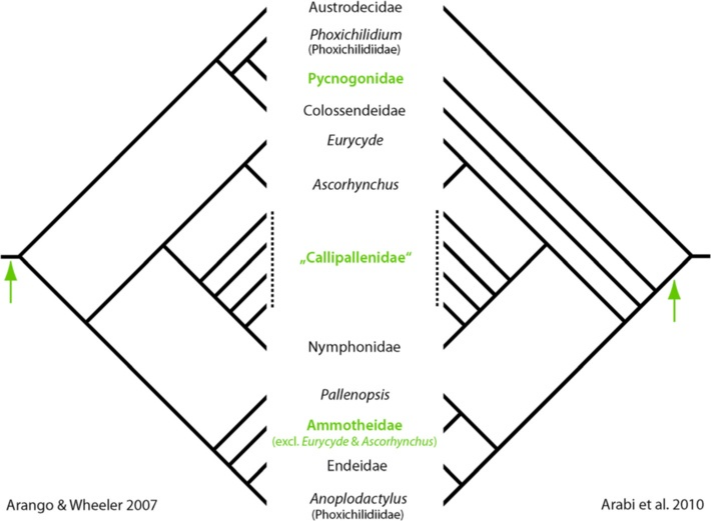
Phylogenetic position of the last common ancestor of the three investigated pycnogonid species. Simplified phylograms of the two most comprehensive phylogenetic analyses of internal pycnogonid relationships (For simplicity, *Nymphon floridanum*, which renders Nymphonidae polyphyletic in Arango and Wheeler (2007), has been omitted). Depending on which hypothesis is favored, the last common ancestor (*green arrows*) of the three investigated species is the stem species of crown-group pycnogonids (Arango and Wheeler 2007, *left side*) or of all extant pycnogonids to the exclusion of Austrodecidae (Arabi et al. 2010, *right side*). The groups highlighted in green include the three species investigated in this study

The discovery of considerable similarities in the SLI pattern of all three species leads to the question of the ancestral pattern of Pycnogonida. A handful of phylogenetic analyses performed mainly during the last decade have sought to resolve the internal phylogeny of extant pycnogonid taxa [63–67]. Yet, whereas some hypotheses based on earlier ideas (e.g. [68, 69]) could be clearly rejected, an uncontested stable pycnogonid phylogeny has still not emerged. For this reason, we here refrain from proposing any of the three species-specific SLI patterns to represent a more ancestral or derived state within crown-group pycnogonids. However, despite the lack of a reliable internal phylogeny, a comparison between the investigated species can nonetheless yield a provisional *minimal* SLI pattern for their last common ancestor, which may have been the stem species of crown-group pycnogonids (depending on which of the phylogenetic hypotheses is favored; see Fig. 10).

If an identified SL-ir neuron type is present in the walking leg ganglia of all three species, it seems reasonable to assume that this neuron has already been present in the walking ganglion of their last common ancestor. Accordingly, we propose that DMNs, cAMN, iAMNs, ALNs, and PLNs have been part of the ancestral repertoire. In cases of intra- and/or inter-specific variations of neuron numbers (iAMNs, ALNs, PLNs), we suggest the lowest consistently observed number to be provisionally put into the ancestral pattern (Fig. 9). A very conspicuous instance of such variation is found in the ALNs, which appear in clusters of variable cell numbers in *P. litorale* but only as single neurons or neuron pairs in *Meridionale* sp. and *C. japonicus*. In line with our minimal approach, we place in this case only a single rather dorsally positioned ALN into the ancestral pattern (Fig. 9).

As the pattern of SL-ir neurons in WLG1 consistently deviates in some aspects from the one in WLG2-4 (see above), the reconstructed ancestral pattern of WLG1 is also slightly different (Fig. 9).

### SL-ir neurons in Euchelicerata versus Pycnogonida

Recent phylogenetic analyses tend to favor Pycnogonida as the sister group to Euchelicerata [e.g. [70–73] , see also [42, 74]). In euchelicerates, the SLI pattern in the VNC has been studied only in few representatives, including spiders (*Cupiennius salei* [75]), opilionids (*Rilaena triangularis* [52]), scorpions (*Pandinus imperator*, *Androctonus australis* [25, 39]) and xiphosurans (*Limulus polyphemus* [25]). In these four taxa, each ventral neuromere gives rise to either one or two clusters of SL-ir neurons with often variable cell numbers, ranging species-specifically from minimally four (*R. triangularis*) to more than 60 (*P. imperator*, *A. australis*). It has been proposed that the euchelicerate stem species featured one anterior and one posterior cluster of SL-ir neurons in each hemiganglion, at least some of the neurons sending contralateral projections into the other ganglion half [10, 26, 40].

Clearly, this putatively ancestral euchelicerate pattern deviates significantly from our observations in pycnogonids. Only in *P. litorale*, a numerically variable, cluster-like arrangement is assessable for the ALNs (with apparently ipsilateral neurites). The remaining array of SL-ir neurons, however, is very similar to the other two investigated pycnogonids, featuring several stereotypic and individually identifiable cells that occur either singly or in pairs.

This pattern disparity between pycnogonids and euchelicerates results in a problematic situation for the reconstruction of the ancestral state in the chelicerate lineage. To resolve this, an out-group comparison might help to achieve character polarization (see below). Nonetheless, however, also critical re-evaluation of the hitherto advocated euchelicerate pattern would be desirable. For instance, the proposed plesiomorphic ‘two-cluster pattern’ with contralateral neurite projections [10, 25] has in fact only been found in the opisthosomal neuromeres of second-stage larvae of *L. polyphemus* [25] (the adult pattern remains still unknown). By contrast, neither the more anterior walking leg-bearing prosomal neuromeres of the same species, nor any ventral neuromere of the other studied adult euchelicerates show a corresponding pattern. Instead, there is only a single cluster per hemiganglion. Accordingly, the reconstructed pattern is solely based on the interpretation of the xiphosuran larval opisthosomal neuromeres as representing the plesiomorphic euchelicerate condition. Deviations from this are interpreted as apomorphic simplifications [25], but a proper phylogenetic analysis is still missing.

Likewise, if taking our pycnogonid data into account, an extrapolation of the variability of SL-ir neuron numbers and the lack of individually identifiable neurons to the entire Chelicerata can be questioned. Even more so, as at least for one euchelicerate representative constant numbers of stereotypic SL-ir neurons are actually reported. In the harvestman *R. triangularis*, exactly four neurons are described per prosomal walking leg hemi-neuromere, all of which with ventro-medial somata and ipsilaterally projecting neurites [52]. Hence, if clusters of variable cell numbers are advocated as being plesiomorphic for Chelicerata, two independent evolution events within the pycnogonid and opilionid lineages would have resulted in individually identifiable SL-ir neurons with largely constant numbers.

### SL-ir neurons in Tetraconata and Myriapoda versus Pycnogonida

Several studies have focused on the comparison and homologization of SL-ir neurons in the VNC of different tetraconate taxa (see, e.g. [10, 24, 36]). As a result, a relatively simple pattern of one anterior and one posterior pair of SL-ir neurons has been suggested for the tetraconate stem species (e.g. [10]). These neurons were proposed to have been unipolar, with ipsilaterally as well as contralaterally extending projections (type B *sensu* Harzsch [25]). In the meantime, studies on additional crustacean groups have demonstrated the presence of additional SL-ir neurons in remipedes and cephalocarids, several of them being found in medial position [26, 62]. As discussed in detail by Stegner and colleagues [62], these findings might necessitate a re-evaluation and extension of the ancestral SLI pattern in Tetraconata.

One key argument for the need of a re-evaluation of the ancestral tetraconate pattern is the situation in Myriapoda, the putative sister group to Tetraconata (=Mandibulata hypothesis; see [42, 73]). The only available study of the SLI pattern in the myriapod VNC revealed a segmental array of nine to twelve neurons (centipedes and millipedes, respectively), the somata of which being arranged singly, in pairs or in groups of four in characteristic positions within the ganglionic cortex [25]. However, apart from one distinct pair of postero-lateral neurons in centipedes that project contralaterally, the course of their neurites could so far not be reliably traced.

Given the SLI pattern disparity between pycnogonids and euchelicerates, an out-group comparison especially to myriapods is one of the necessary next steps when trying to establish character polarization for the chelicerate lineage. Notwithstanding persisting limitations regarding neurite morphology, some noteworthy overall correspondences in the general pattern type can be assessed between pycnogonids and myriapods. These correspondences include (1) a segmental set of SL-ir neuronal somata that are arrayed predominantly singly or in pairs within the cortex, (2) the stereotypic positions of these somata with different lateral and medial as well as anterior to posterior positions, and (3) their similar numbers per hemi-ganglion. Due to these features, several SL-ir neurons can be reliably recognized at the single cell level in different ganglia and across different specimens. This stands in clear contrast to the reconstructed euchelicerate pattern with neuron clusters of variable cell numbers.

Beyond that, however, homologization of single SL-ir neuron types between pycnogonids and myriapods faces the same uncertainties that have been previously encountered in a similar attempt made for cephalocarids and myriapods (see [62]). Preliminary working hypotheses can be discussed, but well-founded homology assumptions remain more often than not elusive. For instance, in all investigated myriapods at least one posterior pair of neurons is present (neuron type ‘e’ *sensu* Harzsch [25]), which – where known – sends out contralateral projections. Obviously, the two distinctive posterior DMNs in the WLG2-4 of pycnogonids with their contralateral projections appear to be promising candidates for homologization. However, in myriapods, the somata of these characteristic posterior neurons are positioned laterally, whereas the pycnogonid DMNs are found far medially. While this difference of soma position need not necessarily imply non-homology, the situation is complicated by pycnogonid walking leg ganglia featuring at the same time the (mostly) paired PLNs with postero-lateral soma position. Yet, as far as we were able to trace their primary neurite, it runs ipsilaterally rather than contralaterally. Accordingly, we are faced with two potential pycnogonid homologues to the myriapod postero-lateral neurons ‘e’, each of them showing only one of two applied indices for homology. To complicate matters even further, myriapods feature an additional neuron type ‘d’ [25], with medial soma that may be shifted posteriorly, but the neurite course of which remains unknown. Apart from the already discussed DMNs, also the less reliably detected VMNs or PVNs of pycnogonids might be considered as potential homologue to this ‘d’ neuron of myriapods.

In our opinion, this amply demonstrates that homologization attempts at the level of SL-ir neuron types is anything but straightforward outside of the tetraconates, and even in the latter taxon more challenging than previously assumed [26, 62]. The here presented pycnogonid data are one of the first steps for a broader data basis in chelicerates (see also [39]). Beyond that, additional investigations on myriapods are still needed to estimate more confidently the true potential of SL-ir neuron patterns in the arthropod VNC within a phylogenetic-evolutionary framework.

### Individually identifiable SL-ir neurons – an ancestral arthropod or panarthropod feature?

Based on evidences from different areas of the CNS, it has been suggested that individually identifiable neurons might represent an apomorphic feature that has evolved only in the mandibulate lineage (e.g. [10, 40]). This view is clearly challenged by the SLI pattern of Pycnogonida. Not only are most of the SL-ir neurons of pycnogonids individually identifiable. Additionally, there are also several overall correspondences between the pattern type of pycnogonids and myriapods. Hence, it appears rather likely that the VNC of the arthropod stem species has already possessed a more stereotypic segmental array of SL-ir neurons. If this should hold true, a pattern of SL-ir neuron clusters with variable cell numbers would have to be considered as being apomorphic either for the entire euchelicerate lineage or at least for some of the euchelicerate taxa.

The original idea of the more variable cluster pattern being plesiomorphic for arthropods found some support from studies on Onychophora [28, 29], which are generally accepted as close arthropod relatives [42]. In these animals, numerous SL-ir neurons are evenly distributed along the two ventral medullary cords, with no indications of segmental arrays, let alone stereotypic individually identifiable cells. Notably, however, it is not beyond doubt whether the gross anatomy of the onychophoran VNC represents a good approximation for the plesiomorphic condition of arthropods, or is rather a highly derived characteristic of the onychophoran lineage itself [30, 76]. In the meantime, two recent studies on Tardigrada – being as well potential close relatives of arthropods – have provided data on the SL-ir neurons in the VNC [30, 31]. Interestingly, both studies point out the presence of few SL-ir neurons in each ventral ganglion, their somata being found in stereotypic positions, although exact locations are unfortunately not described. These new findings may indicate an even earlier appearance of individually identifiable SL-ir neurons already in the panarthropod lineage [30]. To a certain extent, this view depends on whether the ganglionated VNC of tardigrades and arthropods is considered to be homologous [30], or rather interpreted as having evolved two times independently.

## Conclusions

Our study reveals individually identifiable SL-ir neurons in the pycnogonid VNC. As a consequence, the validity of this neuroanatomical feature as apomorphy of mandibulates is challenged. Instead, we propose it to be ancestral for arthropods, if not even for the entire panarthropod lineage. The here presented segmental pattern of SL-ir neurons of pycnogonids deviates significantly from the one reported for the euchelicerates that have been studied so far. In order to better resolve and understand the evolution of SL-ir neurons in the latter lineage, partial reinvestigation and a denser sampling of different representatives is desirable. By contrast, the overall similarities between the pycnogonid and myriapod patterns may be indicative of single cell homologies in these two taxa. However, this notion awaits further substantiation from future studies.

## Additional files

Additional file 1:
**Serotonin-like immunoreactive neurons and neuropilar domains in walking leg ganglion 2 of** ***Pycnogonum litorale*** **.** Acetylated α-tubulin (red) and serotonin-like immunoreactivity (white), Imaris volume (Maximum intensity projection). Clipping planes have been applied to the ventral ganglion surface to improve the view on the segmental set of SL-ir neurons. The movie starts in ventral view. The ganglion rotates briefly (counterclockwise) around the antero-posterior-axis, then turns back into ventral view. Upon masking of the tubulin signal, the ganglion is zoomed in. Note that the two DMN somata are of about the same size. Only a single PLN is labeled in the right hemiganglion, whereas two PLNs are visible in the left (one with low signal intensity). The cAMN soma is positioned far ventral, the one of the left hemiganglion being found exceptionally far lateral. The neurite of the right cAMN shows the typical ipsilateral projection and subsequent contralateral loop, extending through the median SL-ir domain into the other body half. The loose arrangement of ALNs encompasses ventral as well as far dorsal somata, their neurites being only weakly labeled but extending without exception into the lateral region of the ipsilateral SL-ir neuropilar domain. Note posteriorly in the cortex very weakly labeled somata, presumably belonging to the unreliably identifiable VMN and PVNs. Additional file 2:
**Serotonin-like immunoreactive neurons and neuropilar domains in walking leg ganglion 3 of** ***Meridionale*** **sp.** Serotonin-like immunoreactivity (glow), Imaris volume (Maximum intensity projection). To improve the view on the segmental set of SL-ir neurons, the unspecifically stained neural sheath (compare to Fig. 4b) has been virtually masked (Imaris Contour Surface). The movie starts in ventral view. The ganglion is rotated and zoomed in and out, in order to present better views of the different neuron types. Note that the two DMN somata are of different size and that the contralateral neurite of the smaller DMN passes slightly anterior to the thick neurite of the large DMN. Two PLNs are labeled, one showing distinctly lower signal intensity. In the left hemiganglion, the soma of the weakly labeled PLN is positioned untypically far medial. Only two ALNs are present per body half (one weakly labeled), lying in the left body half further apart than in the right. The cAMN soma is found very close to one of two strongly labeled iAMN somata. In left hemiganglion, the cAMN soma is not fully included in the scan, but the characteristic contralateral loop of its neurite is clearly visible. The ispilateral projections of the intensely labeled ventral iAMNs extend far lateral into the anterior portion of the SL-ir domain. Additionally, two weakly stained iAMN somata are visible in each hemiganglion. Note complete absence of VMN and PVNs. Additional file 3:
**Serotonin-like immunoreactive neurons and neuropilar domains in walking leg ganglion 2 of** ***Cilunculus japonicus*** **.** Serotonin-like immunoreactivity (glow), Imaris volume (Blend). The movie starts in ventral view. The ganglion is rotated and zoomed in and out, in order to present better views of the different neuron types. Annotations of the latter appear successively in the course of the movie. Note distinct size differences between the two DMN somata of each hemiganglion. The contralateral neurite of the large DMN are clearly visible, the more slender neurite of the smaller DMN can be only followed in the left body half. While two PLNs are labeled in the left hemiganglion (one showing distinctly lower signal intensity), only one is stained in the right. Note the presence of two relatively weakly labeled PVNs per hemiganglion. Two weakly labeled vALNs are found in each hemiganglion, being set off from one dALN in the right and two dALNs in the left body half. The cAMN soma is found very close to one of two strongly labeled iAMN somata. The cAMN soma is located far ventral, the contralateral loop of its neurite being difficult two follow in this scan. Three or four iAMN somata are discernible (right and left hemiganglion, respectively). From some of those, the ipsilaterally extending neurite can be seen (e.g. annotated iAMN soma). Note also the presence of one VMN in each hemiganglion. Additional file 4:
**Ventral serotonin-like immunoreactive neurons in walking leg ganglion 2 of** ***C. japonicus*** **.** SLI in the ventral ganglion half shown in inverted b/w image for better contrast. Dashed line indicates midline region. Note weak signal intensity of vALNs and PVNs. Small arrows follow the ipsilaterally extending VMN neurite. Asterisk marks the weakly labeled neurite of a PVN. Large arrowheads highlight an anteriorly projecting branch of the contralateral DMN (soma and primary neurite not in section). Small arrowheads trace the course of the contralaterally extending cAMN neurite. Additional file 5:
**Detail of two bipolar cAMNs in walking leg ganglion 3 of a** ***P. litorale*** **specimen.** SLI signal shown in inverted b/w image for better contrast. Dashed line indicates midline region. In addition to the strongly labeled typical neurite leaving each soma (arrowheads) slender projections extend directly towards the SL-ir median domain (small arrows).

## Abbreviations

ALN: Antero-lateral neuron
dALN: Dorsal antero-lateral neuron
vALN: Ventral antero-lateral neuron
AMN: Antero-medial neuron
cAMN: Contralateral antero-medial neuron
iAMN: Ipsilateral antero-medial neuron
apc: Apical cell cluster
br: Brain
CNS: Central nervous system
cut: Cuticle
con: Connective
DMN: Dorso-medial neuron
GLN: Glomerulus-like neuropils
Hoe: Hoechst nuclear staining
lmb: Longitudinal muscle bundle(s)
mg: Midgut
np: Neuropil
PLN: Postero-lateral neuron
pvc: Postero-ventral commissure
PVN: Postero-ventral neuron
seg: Subesophageal ganglion
SLI: Serotonin-like immunoreactivity
SL-ir: Serotonin-like immunoreactive
snv: Segmental nerve
tub: Anti-acetylated alpha-tubulin immunolabeling
vlt: Ventral longitudinal tract
VMN: Ventro-medial neuron
VNC: Ventral nerve cord
wlg x: Walking leg ganglion x
wln1: Walking leg neuromere 1
