# Supplementary figures and images for: Serotonin-immunoreactivity in the ventral nerve cord of Pycnogonida – support for individually identifiable neurons as ancestral feature of the arthropod nervous system
Source: BMC Evol Biol. 2015 Jul 10;15:136. doi: 10.1186/s12862-015-0422-1 (PMC4496856; doi:10.1186/s12862-015-0422-1)

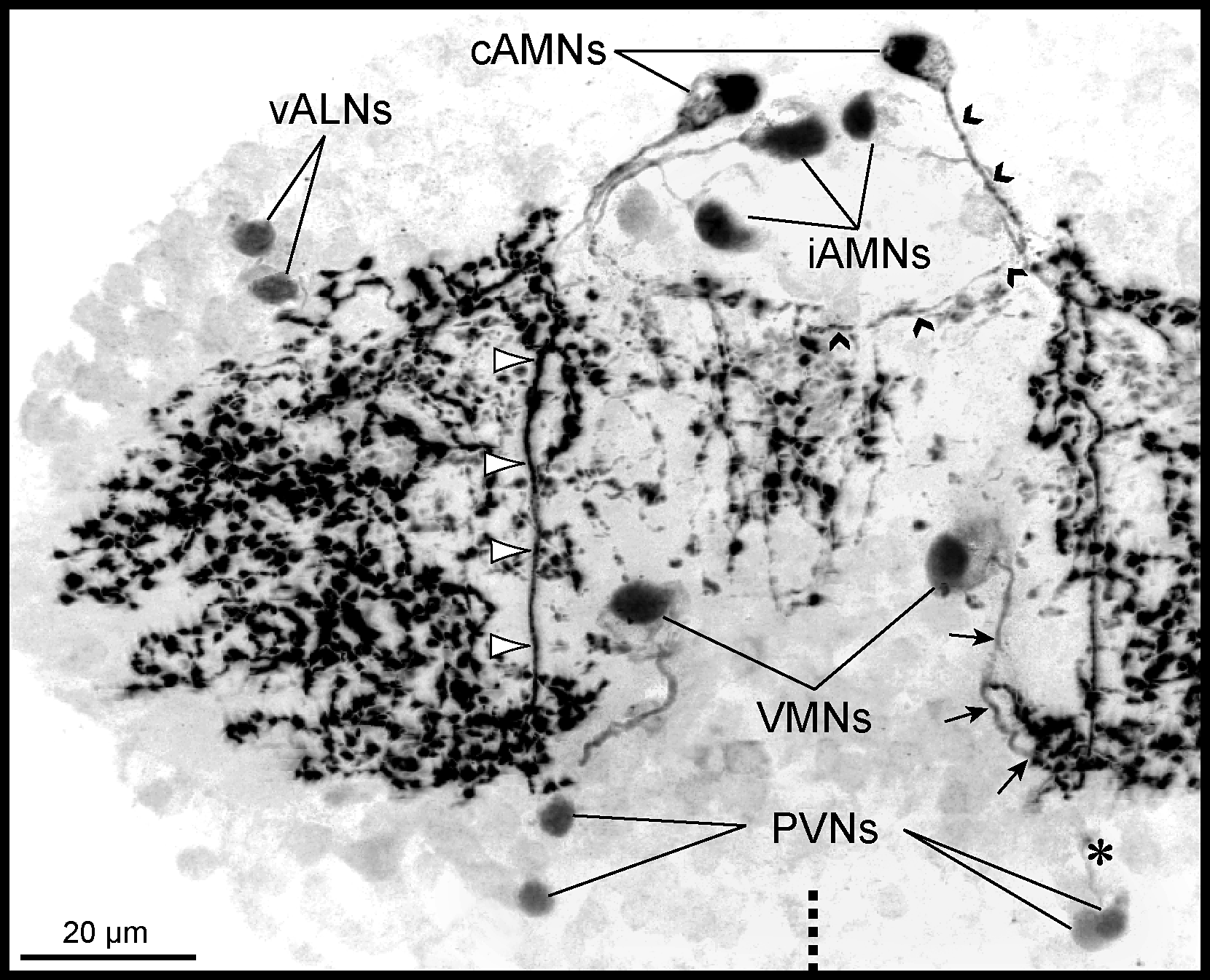

Supplement: Additional file 4: — Ventral serotonin-like immunoreactive neurons in walking leg ganglion 2 of C. japonicus . SLI in the ventral ganglion half shown in inverted b/w image for better contrast. Dashed line indicates midline region. Note weak signal intensity of vALNs and PVNs. Small arrows follow the ipsilaterally extending VMN neurite. Asterisk marks the weakly labeled neurite of a PVN. Large arrowheads highlight an anteriorly projecting branch of the contralateral DMN (soma and primary neurite not in section). Small arrowheads trace the course of the contralaterally extending cAMN neurite. [file 12862_2015_422_MOESM4_ESM.tif]

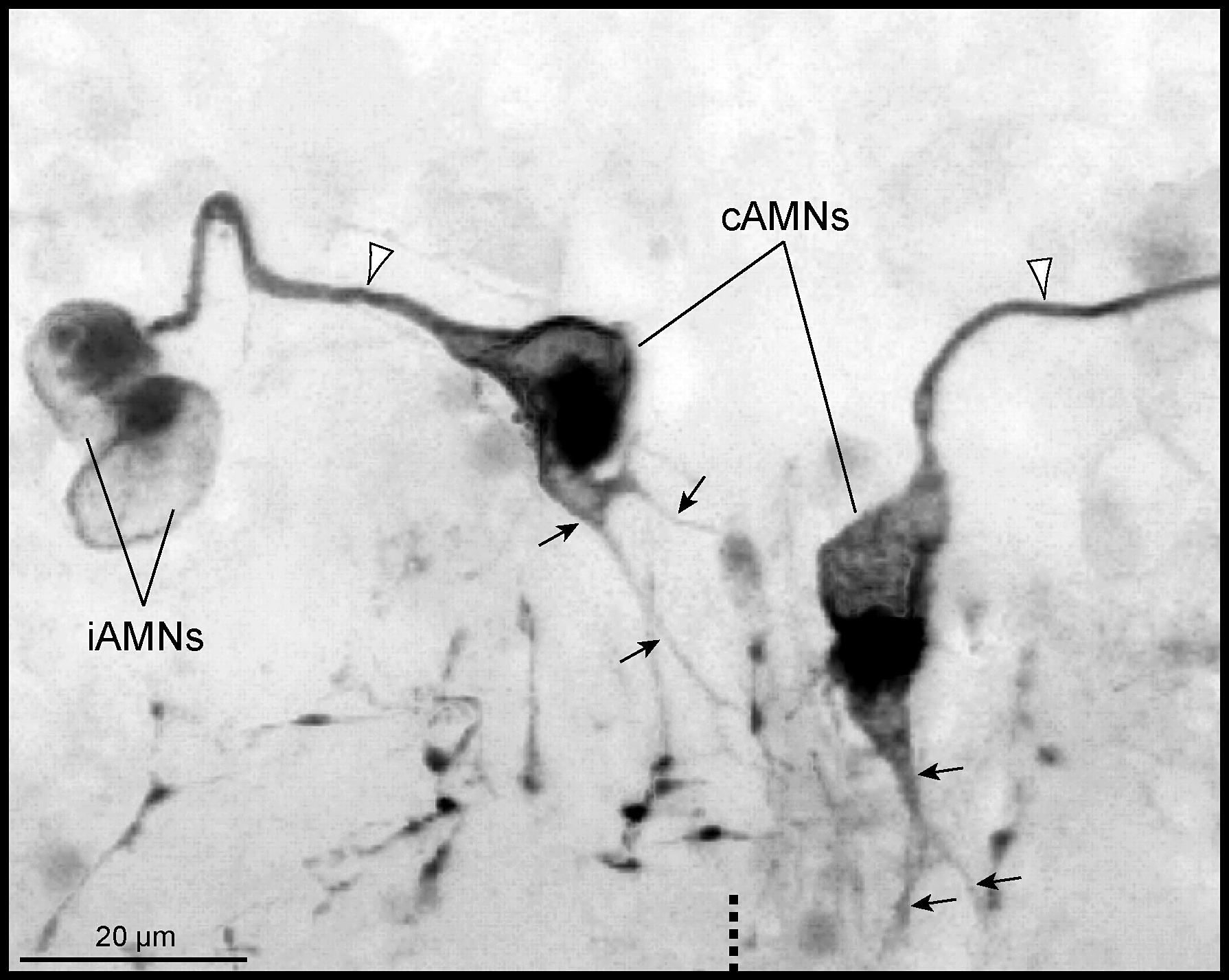

Supplement: Additional file 5: — Detail of two bipolar cAMNs in walking leg ganglion 3 of a P. litorale specimen. SLI signal shown in inverted b/w image for better contrast. Dashed line indicates midline region. In addition to the strongly labeled typical neurite leaving each soma (arrowheads) slender projections extend directly towards the SL-ir median domain (small arrows). [file 12862_2015_422_MOESM5_ESM.tif]
